# Influence of the Ozonation Process on Expanded Graphite for Textile Gas Sensors

**DOI:** 10.3390/s25175328

**Published:** 2025-08-27

**Authors:** Paulina Rzeźniczak, Ewa Skrzetuska, Mohanapriya Venkataraman, Jakub Wiener

**Affiliations:** 1 Faculty of Material Technologies and Textile Design, Textile Institute, Lodz University of Technology, ul. Żeromskiego 116, 90-924 Łódź, Poland; paulina.szablewska@dokt.p.lodz.pl; 2 Department of Material Engineering, Faculty of Textile Engineering, Technical University in Liberec, Studentská 1402/2, 461 17 Liberec 1, Czech Republic; mohanapriya.venkataraman@tul.cz (M.V.); jakub.wiener@tul.cz (J.W.)

**Keywords:** ozonation, expanded graphite, textile gas sensors

## Abstract

In view of the growing demand for flexible, conductive and functional materials for textile gas sensor applications, the effects of ozonation on the properties of expanded graphite (EG) in textile structures were analyzed. Four types of fabrics (cotton, polyamide, viscose, para-aramid) coated with pastes containing EG, which had previously been subjected to a 15-min and 30-min ozonation process, were examined. The paste was prepared using Ebecryl 2002 and the photoinitiator Esacure DP250 and then applied by screen printing. Surface resistance, scanning microscopy and wetting angle analyses were performed. The results showed that short-term ozonation (15 min) notably improved the electrical conductivity and adhesion of EG to the textile substrate, while longer exposure (30 min) led to deterioration of the conductive properties due to excessive functionalization and fragmentation of the conductive layer. The lowest surface resistance was observed in the sample subjected to 15 min of ozonation. The conclusions indicate that a properly controlled ozonation process can increase the usability of EG in sensor applications, especially in the context of smart clothing; however, the optimization of the modification time is crucial for maintaining the integrity and durability of the conductive layer.

## 1. Introduction

One of the most versatile and widespread elements on Earth is carbon. It plays a key role in organic chemistry and biology. It has the unique ability to form stable bonds with other carbon atoms and other elements, which are the basic building blocks of life. And this element is characterized by its ability to occur in various allotropic forms. These forms differ in crystal structure and physical properties despite being composed of the same atoms. We can distinguish allotropic forms of carbon, such as diamond, graphite, fullerenes, carbon nanotubes and graphene.

- Diamonds are known as the hardest natural material. It has a three-dimensional structure built by bonding one carbon atom with four other atoms, resulting in a very hard and durable crystal lattice.
- In graphite, carbon atoms form hexagonal planes that can easily slide relative to each other, giving it a layered structure. Graphite has conductive properties.
- Fullerenes are molecules with a spherical or ellipsoidal shape, formed by carbon atoms bonded together.
- Carbon nanotubes are characterized by high electrical conductivity, and their cylindrical structure exhibits very high mechanical strength.
- Graphene is thin and light, yet durable and an excellent conductor of electricity. Its structure is a single layer of carbon atoms arranged in a honeycomb pattern.

The variety of carbon allotropic forms means that it is used in many fields—from the jewelry industry, through electronics, to nanotechnology. Understanding the properties of these structures is fundamental to the development of modern technologies [1,2,3,4,5]. In this article, the authors will focus in detail on one allotropic form, which is graphite.

**Figure 1:**
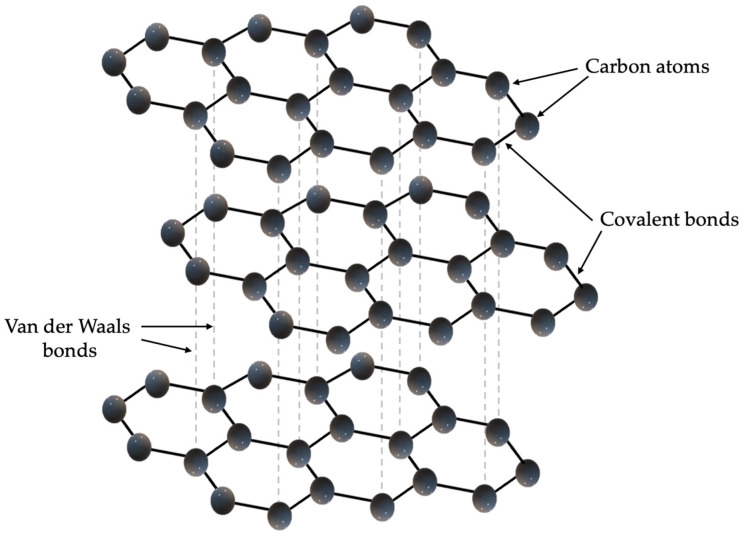
Three-dimensional diagram of graphite layers of carbon atoms.

As previously described, graphite has a layered structure, and each carbon atom is bonded to three others in a plane, which is shown in Figure 1. The layers, which can also be called graphene planes, are also bonded together by weak Van der Waals bonds, allowing they can slide relative to each other. Graphite is dark grey to black in color, has a metallic luster, is soft and cleavable, has a density of 2.2 g/cm^3^ and a melting point above 3600 °C. Graphite is characterized by very good electrical conductivity, reaching approximately 10^4^ S/m in the direction parallel to the graphene planes, and high thermal conductivity, which can reach up to 2000 W/m·K (in the direction of the planes), making it one of the best natural thermal conductors. However, it should be noted that both electrical and thermal conductivity are highly anisotropic—in the direction perpendicular to the planes, these values are significantly lower (e.g., thermal conductivity drops to approximately 6 W/m·K) [6]. Graphite can be obtained naturally by mining graphite ores, and synthetically from carbon precursors at high temperatures (around 3000 °C) during the pyrolysis and graphitization process.

**Figure 2:**
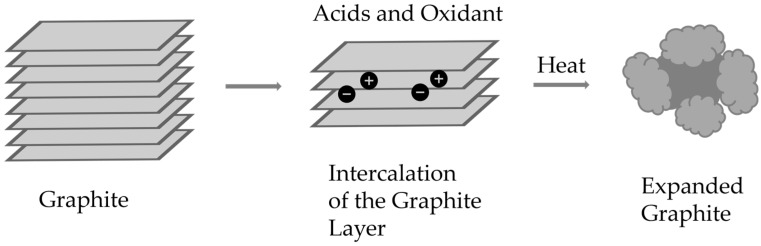
Diagram of the formation of expanded graphite.

The research described in this article used expanded graphite. This is a form of graphite that has been chemically modified. Through intercalation, i.e., introducing chemical compounds between the graphite planes, and then rapidly heating the graphite layers expand, resulting in a porous and volumetrically enlarged material. The whole process looks like this: graphite is subjected to the action of a mixture of acids and oxidants, which penetrate between its layers. After drying, the graphite is subjected to rapid heating at a high temperature. This causes rapid evaporation of the introduced particles and an “explosion” of the structure. As a result, expanded graphite is created with a volume several hundred times larger and a highly developed surface area. The process is shown in Figure 2. Expanded graphite is characterized by very low density, large specific surface area, high chemical and thermal resistance, very good sorption properties and good thermal and electrical conductivity. Table 1 presents a comparison of the properties of graphite and expanded graphite [7,8,9,10].

The ozonation process was used to modify expanded graphite. Ozone (O_3_) is defined as an allotropic form of oxygen, consisting of three oxygen atoms. It is characterized by a pungent odor and is a very reactive oxidant. It can break chemical bonds in organic and inorganic molecules [11,12,13,14]. Ozonation is a process in which the molecules it reacts with are oxidized or decomposed. It is created by corona discharge or UV radiation:

(1) $$3O_{2}+energy\to 2O_{3}$$

The chemical reaction of ozone with expanded graphite is an oxidation process that occurs on the surface of graphene layers (i.e., expanded graphite). It is a physicochemical reaction that modifies the structure and properties of graphite, but it does not occur in a simple way like a classic stoichiometric reaction—that is why it is not written as one simple summary reaction but as several reactions. We can distinguish the following:

1. Ozone adsorption, where ozone attaches to the surface of graphite layers.
2. Oxidation, where ozone molecules react with carbon atoms, especially those at defects, edges or interlayers of graphite. Functional groups are formed:
  - epoxy (–C–O–C–)
  - hydroxyl (–OH)
  - carboxyl (–COOH)
  - carbonyl (C=O)
3. Formation of graphene oxide: Due to intensive oxidation of expanded graphite, graphene oxide (GO) can be formed.

Schematic reaction:

(2) $$C_{n}+O_{3}\to C_{n}O_{x}(functional\;groups)$$

In words: graphite + ozone → oxidized graphite with oxygen groups

The general effects of the ozonation reaction are as follows:

- Increased hydrophilicity (greater affinity for water)
- Reduced electrical conductivity
- Possibility of further chemical modifications
- Opening of the graphite layer structure [15,16,17,18].

Textronics is an interdisciplinary field of science and technology that combines textile materials with modern electronics, microsystems and intelligent materials. The aim of this technology is to integrate electronic components, such as sensors, cables, energy cells or signal processing systems, directly with textile materials, while maintaining their functional, mechanical and wearing comfort properties. The development of textronics is very strong currently and fits into the global trend of miniaturization of electronics and the growing demand for intelligent wearable systems. Textronic solutions are used, among others, in medicine, sports, rescue, military and environmental monitoring. Examples of practical applications include textile electrodes for measuring bioelectric signals (e.g., ECG, EMG), conductive threads for data transmission, heating fabrics or even autonomous power systems based on solar or thermoelectric cells integrated with clothing. Of particular importance in this field are carbon materials (e.g., graphene, carbon nanotubes, expanded graphite), conducting polymers (e.g., PEDOT:PSS, polyaniline) and layered printing and coating technologies [19,20,21,22]. One of the most dynamically developing directions is textronic gas sensors, which allow for continuous, low-intrusive detection of toxic or harmful chemical compounds in the user’s environment. The need for their development results from both occupational safety requirements, e.g., in the chemical industry and domestic environments, and the growing interest in environmental health in the context of cities and climate change [23].

In recent years, special attention has been paid to the use of nanomaterials with high specific surface area and unique electronic properties, such as graphene, metal oxides, MXenes or conductive polymers, e.g., PEDOT:PSS, PANI, which can be deposited on textile substrates using printing, immersion or electrospinning methods [22,24]. These hybrid materials enable the construction of flexible, breathable and conductive structures that respond to the presence of gases such as NO_2_, NH_3_, CO or VOCs, i.e., volatile organic compounds, often at concentrations of parts per billion (ppb) [25]. An example of such a solution is a textile sensor based on the core-shell architecture, using PVDF/PZT fibers covered with a PANI/PVP coating, capable of simultaneously detecting NO_2_ concentration and mechanical pressure [24]. In turn, a review of the current state of knowledge emphasizes the increasing effectiveness and stability of gas sensors built into textiles, while pointing out technological barriers such as moisture resistance, signal repeatability, selectivity and compatibility with everyday use (e.g., washing, bending) [22,23].

The development of such sensors is a significant step towards creating smart clothes of the future, capable of independently collecting and processing environmental data in real time, which is of particular importance in medical, rescue, military and civilian air quality monitoring systems. The research discussed in this article aims to investigate the potential use of expanded graphite in textile-based textronic systems for gas sensors, to analyze the effect of the ozonation process and to compare the obtained properties.

## 2. Materials and Methods

Four textile materials were used to fabricate the textronic gas sensors for the research described in this article—cotton knit, polyamide knit, viscose fabric and para-aramid fabric. The materials selected differed in both composition and structure. Table 2 below presents some of the parameters of selected textile materials. The surface mass, thickness and air permeability are presented. These parameters are important in the context of the subsequent application of the textile sensor, whether in clothing or household products.

Two durations of ozonation were carried out on samples weighing 1 g of expanded graphite produced by scientists at the Technical University of Liberec. The research setup consisted of a pump that injected pure ozone into a closed flask containing graphite and a flowmeter, enabling determination of the amount of ozone consumed in the reaction with graphite. All components were connected by tubes in a linear manner. The pump injected 3 L of pure ozone per minute, approximately 30 mg/L. Processes lasting 15 min and 30 min were carried out. During the process, the meter at the end of the system indicated a flow of 25 mg/L. This means that 5 mg/L was absorbed by the surface of the expanded graphite. In this way, expanded graphite was created after the 15-min ozonation process and after the 30-min process, for used in producing printing pastes.

These materials were modified with three pastes made of water, ebecryl, esacure, expanded graphite and expanded graphite after the ozonation process in a ratio 79:10:10:1 for each component. Based on the analysis of information on the agents, an aliphatic urethane acrylate was selected as the binder, which cross-links under the influence of UV radiation in the presence of a photoinitiator. The aliphatic urethane acrylate was used as the Ebecryl 2002 product from Cytec (Östringen, Germany), and the Esacure DP250 product from Lamberti (Gallarate, Italy) was used as the photoinitiator. The choice of Ebecryl 2002 was dictated by the information that it is used as a binder for inkjet inks and in the pigment dyeing process on cotton, viscose, wool and polyester substrates. Its molar mass is 2500 g/mol, viscosity in aqueous solution is 25,000 mPa·s, measured at 25 °C, and density is 1.1 g/cm^3^. Esacure DP 250 is a non-ionic agent, a photoinitiator used in UV-initiated polymerization in aqueous solutions. This compound is a stable aqueous emulsion based on approximately 32% of a mixture of active photoinitiators such as the following:

- 2,4,6-trimethylbenzoyldiphenylphosphine oxide
- α-hydroxyketones
- benzophenone derivatives.

**Figure 3:**
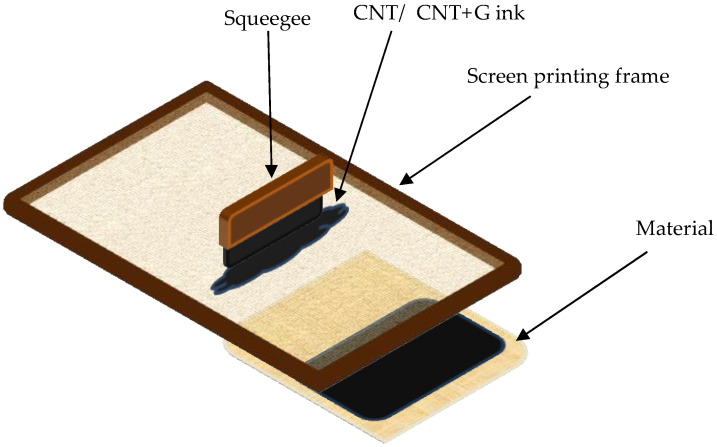
Screen printing modification of textile materials by spreading the printing paste onto the material through a screen printing frame using a squeegee [26].

After adding all the ingredients to the beaker were stirred by a magnetic stirrer for 30 min. This helped to distribute the expanded graphite throughout the paste. Expanded graphite is characterized by low mass and high volatility. After adding all the ingredients to one vessel, it tended to float at the top of the liquid. After mixing the paste, it was also exposed to ultrasound for 30 min at 70 W and 20 kHz. After this process, the particles of all the ingredients were strongly broken down and mixed, resulting in a uniform paste suitable for the printing process. Textile materials were modified with pastes using screen printing technology. Figure 3 schematically shows how this process was carried out. A screen was placed on the textile material, through which a paste with expanded graphite was printed with a squeegee. The modification was made on a 60 T polymer mesh from SATTI with an aluminum frame. The mesh thread diameter was 0.0026 or 66.0 μm. The open area was 37.8%.

## 3. Results

After the paste had bonded with the material and the moisture had evaporated, the analysis of the resulting textronic solutions began.

### 3.1. Surface Resistance

**Figure 4:**
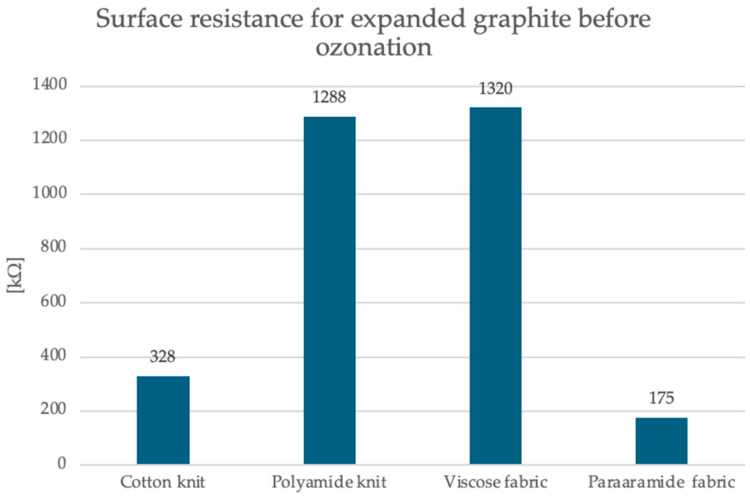
Graphical representation of surface resistance results for printed textronic systems with expanded graphite before the ozonation process.

**Figure 5:**
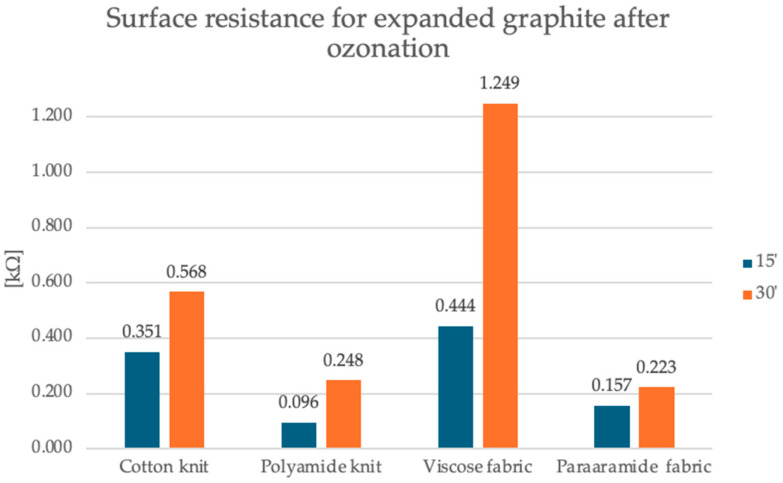
Graphical representation of surface resistance results for printed textronic systems with expanded graphite after the 15- and 30-min ozonation processes.

To carry out measurements in accordance with the standard PN-EN1149-1 [27], ring electrodes with constant dimensions and constant voltage were used. Ten measurements were carried out for each manufactured textronic system under standard conditions. The study was conducted to assess the surface resistance of the developed textronic systems for their potential as gas sensors. The gas sensors in this article are based on changes in surface resistance depending on the concentration of hazardous gases in the environment. This parameter should have values that allow for analysis of changes in the MΩ range. The graphs in Figure 4 and Figure 5 show the results of the surface resistance test. The results are also presented in Table 3.

The first graph shows the surface resistance values for the expanded graphite layer on four different substrates: cotton knit fabric, polyamide knit fabric, viscose knit fabric and para-aramid knit fabric. It is observed that the para-aramid fabric had the lowest resistance, and its value was 174 kΩ, while the cotton knit fabric showed a moderate value of 120 kΩ. The highest values were recorded for polyamide knit fabric and viscose knit fabric, and they were 1288 kΩ and 1320 kΩ, respectively, which suggests weaker conductivity or worse adhesion of the graphite layer to these materials. The second graph shows data for the same four materials after 15 and 30 min of the ozonation process. All samples show a radical decrease in surface resistance—even by several orders of magnitude. After 15 min of ozonation, the resistance values range from 0.950 kΩ for the polyamide knit fabric to 0.444 kΩ for the viscose fabric. After 30 min, the surface resistance values are still much lower compared to the samples with expanded graphite before the ozonation process, but slightly higher compared to the samples after the 15-min ozonation process for most materials. In this case, the resistance values range from 0.223 kΩ for the para-aramid fabric to 1.249 kΩ for the viscose fabric. The polyamide knit fabric achieved a result of 0.248 kΩ, and the cotton knit fabric 0.568 kΩ.

### 3.2. Scanning Microscopy

**Figure 6:**
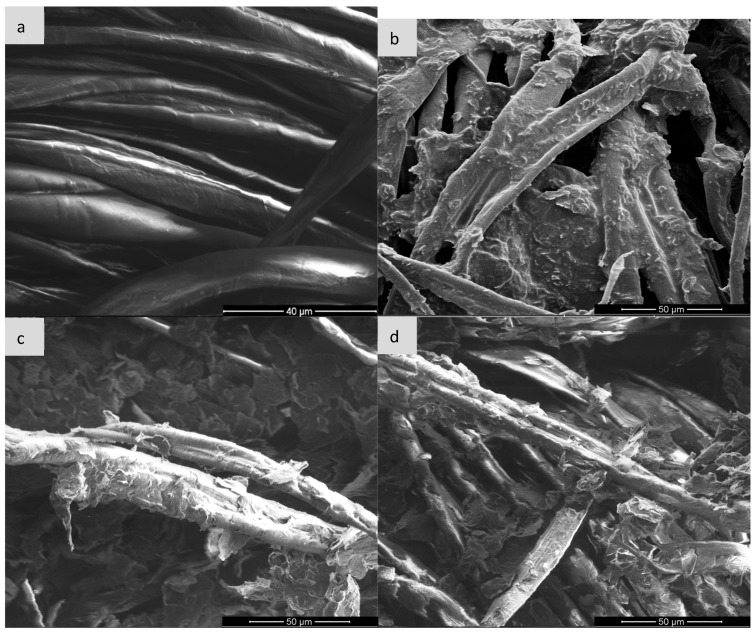
Comparison of scanning microscope images for cotton knitwear: (**a**) pure sample, (**b**) sample with expanded graphite paste, (**c**) sample with expanded graphite paste after 15 min of ozonation, (**d**) sample with expanded graphite paste after 30 min of ozonation.

**Figure 7:**
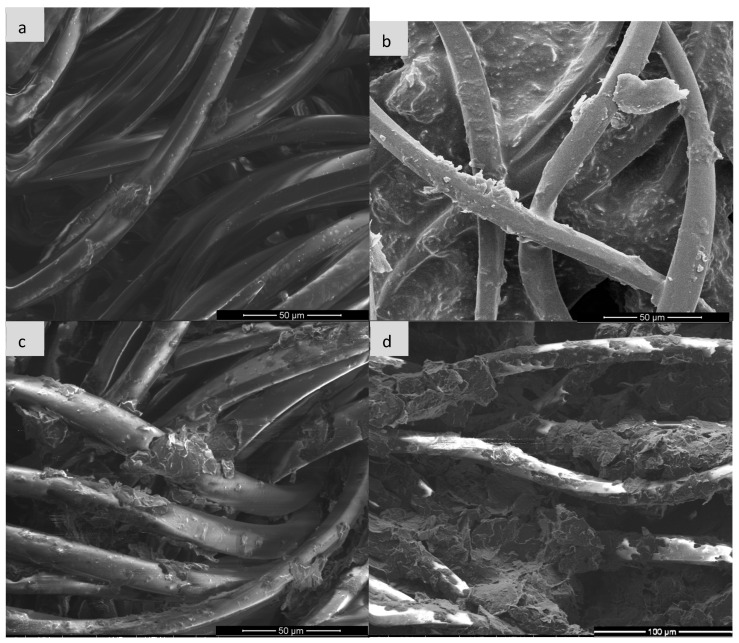
Comparison of scanning microscope images for polyamide knitted fabric: (**a**) pure sample, (**b**) sample with expanded graphite paste, (**c**) sample with expanded graphite paste after 15 min of ozonation, (**d**) sample with expanded graphite paste after 30 min of ozonation.

**Figure 8:**
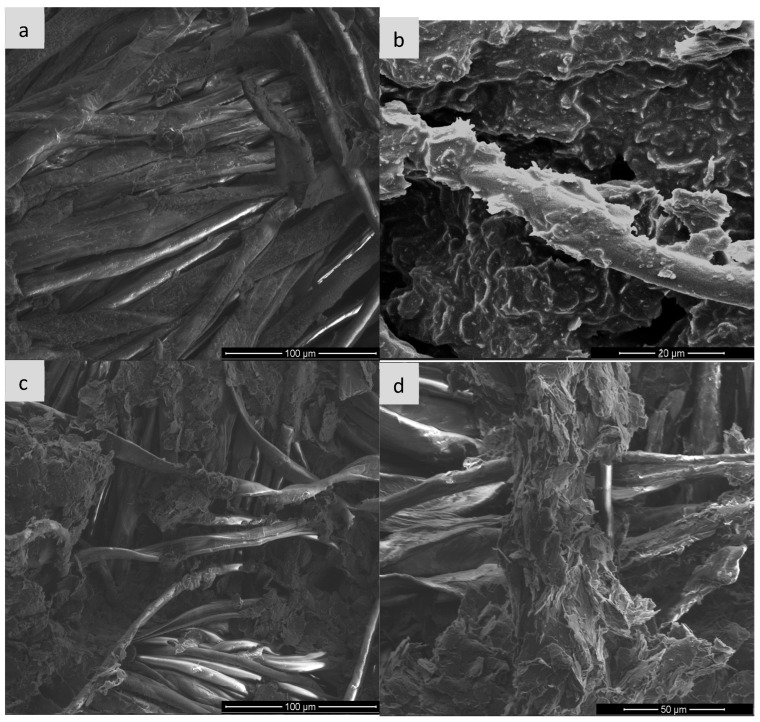
Comparison of scanning microscope images for viscose fabric: (**a**) pure sample, (**b**) sample with expanded graphite paste, (**c**) sample with expanded graphite paste after 15 min of ozonation, (**d**) sample with expanded graphite paste after 30 min of ozonation.

**Figure 9:**
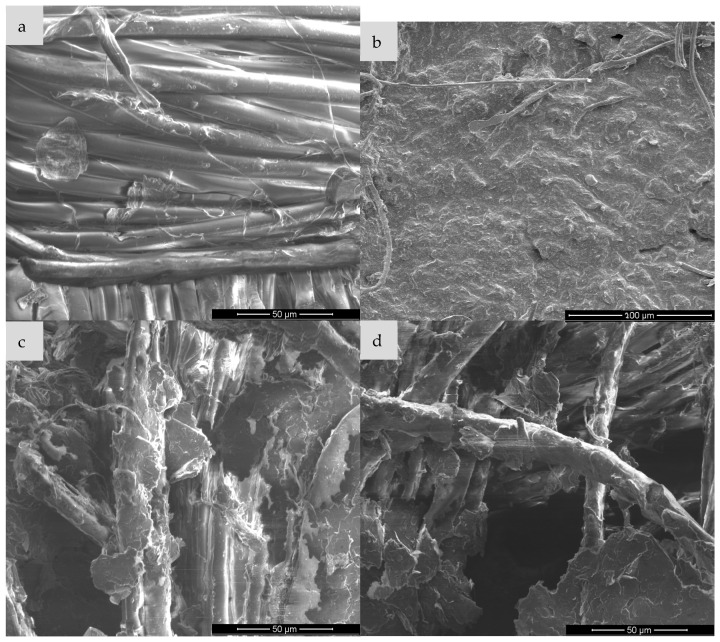
Comparison of scanning microscope images for para-aramid fabric: (**a**) pure sample, (**b**) sample with expanded graphite paste, (**c**) sample with expanded graphite paste after 15 min of ozonation, (**d**) sample with expanded graphite paste after 30 min of ozonation.

Scanning electron microscopy (SEM) is one of the basic techniques for characterizing materials at the micro- and nanoscale level, enabling detailed assessment of the morphology, topography and surface structure of the sample. It is often used to analyze polymer composites, functionalized fabrics or conductive materials such as expanded graphite (EG). In the context of expanded graphite (EG) research, SEM enables visualization of the degree of fabric coverage (e.g., cotton, polyamide), assessment of the homogeneity of the EG layer before and after ozonation, identification of micro-defects, cracks or aggregates, and allows for linking visual observations with surface resistance measurements. Such analysis is particularly important for optimizing surface modification processes and analyzing the relationship between structure and conductive properties [28,29,30] and for this purpose, images were taken for each type of textronic system produced. The images were taken on equipment available at the University of Technology in Liberec and the University of Technology in Lodz (FEI company, Hillsboro, OR, USA). Figure 6, Figure 7, Figure 8 and Figure 9 below show images obtained during examination with a scanning electron microscope, sorted by textile materials.

Image (a) shows the structure of cotton fibers without any modification. The fibers are smooth, without obvious deposits on the surface, and their arrangement is orderly. Characteristic grooves and twists typical of cellulose natural fibers are visible. After the EG application (image b), the surface of the fibres is covered with an irregular, rough layer of graphite paste. The presence of material deposits and their partial adhesion to the fabric surface is observed. However, the structure of the coating is not uniform—there are places of exposed fibres. Ozonation of the sample with EG for 15 min leads to a significant increase in wettability and adhesion of the graphite layer, as seen in image c. The layer covering the fibres becomes more compact, and the material adheres better to the surface. A significant reduction in agglomeration and improvement in the uniformity of the EG distribution can be seen. The surface seems to be more “integrated” with the textile substrate. With the ozonation time extended to 30 min, the surface still shows good contact with the fibres, but signs of excess oxidation appear—in the form of fragmentation and possible flaking of the EG layer as seen in image d. A greater number of cracks and micro-damages can be observed in the conductive layer, which may suggest degradation of the material due to too long exposure to ozone.

The analysis of SEM images in Figure 7 allows for the assessment of morphological changes in the surface of the polyamide knitted fabric resulting from the application of the paste containing expanded graphite, before and after the ozonation process. The reference sample (a), containing no additives, is characterized by a uniform and smooth fiber surface and an ordered structure, typical of a pure polymer material. In sample (b), on which the paste with non-ozonated expanded graphite was applied, irregular particle deposits are visible on the fiber surface, which leads to a significant increase in their roughness and partial blurring of the contours of individual fibers. In turn, sample (c), in which the paste containing expanded graphite ozonated for 15 min was used, shows a smaller amount of deposit on the surface—the paste layer is thinner and less compact, and individual fibers become more visible. This may indicate a partial modification of the graphite structure due to ozonation, affecting its ability to create a coherent layer on the surface of the knitted fabric. Sample (d), with graphite paste ozonated for 30 min, shows a further reduction in the amount of material deposited on the fibres and visible exposure of their surface. Local unevenness and delamination are also observed, which may result from the enhanced chemical modification of graphite affecting the rheological properties of the paste and its interactions with the polymer substrate.

Analysis of the above images allows for the assessment of the effect of the paste containing expanded graphite (ozonated and non-ozonated) on the surface structure of the viscose fabric. The clean sample (a) presents a structure typical of viscose fibres—the surface of the fibres is smooth, with a small number of natural irregularities, and their arrangement is relatively ordered. In sample (b), in which the paste with non-ozonated expanded graphite was used, a significant coverage of the fibres with a layer of filling material is observed. The paste forms a compact, adherent layer, which in places covers the fibres almost completely, limiting their visibility. The surface seems more compact and devoid of porosity, which indicates good adhesion of the paste to the fibres. In sample (c), in which the paste containing expanded graphite subjected to 15-min ozonation was used, a partial reduction in the compactness of the coating is visible—the paste covers the fibres less evenly, and their contours are more visible. It can be assumed that ozonation affected the structure of graphite, limiting its ability to form a compact layer on the surface of the fibres. In turn, sample (d), in which graphite ozonated for 30 min was used, shows the most advanced changes—the paste occurs in the form of porous, loosely bound aggregates and the fibres are almost completely exposed. There are also visible signs of mechanical delamination and cracks, which may be the effect of chemical interaction between the ozonation product and the viscose fibres.

Figure 9 shows SEM micrographs of the surface of the para-aramid fabric in the pure state and after modification with expanded graphite paste—both non-ozonated and ozonated for different times. The reference sample (a) is characterized by a highly ordered, compact structure of para-aramid fibers, with well-defined boundaries between individual filaments. The surface is relatively smooth, and the presence of sporadic defects indicates a typical microstructure of a technical material with high mechanical resistance. In sample (b), on which the paste with non-ozonated expanded graphite was applied, almost complete coverage of the fibers with a homogeneous, compact layer of the filling material is observed. A clear loss of the clarity of the fiber structure is visible, which indicates good adhesion of the paste to the substrate. In the case of sample (c), in which the paste containing graphite ozonated for 15 min was used, significant changes are already visible—the graphite layer becomes more porous and irregular, and in some places the fibres are partially exposed. The observed loosening of the structure may result from chemical modification of the graphite surface, reducing its cohesion and interaction with the fiber. In sample (d), with graphite ozonated for 30 min, this effect is even more intense: the surface of the material shows significant morphological differentiation, and the fibres are almost completely visible, surrounded only by the remains of dispersed fragments of the paste. Additionally, local defects and delamination are noticeable, which may indicate deterioration of the adhesive properties of the paste and reduced compatibility with para-aramid fibres because of intensive ozonation of the graphite.

### 3.3. Contact Angle

**Figure 10:**
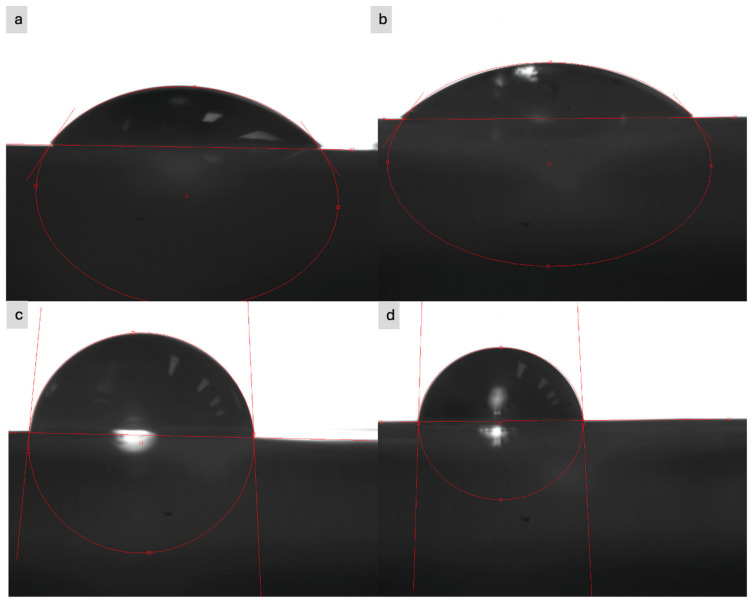
Presentation of example images obtained from an optical microscope: (**a**) cotton knit fabric with a drop of water, (**b**) polyamide knit fabric with a drop of water, (**c**) viscose fabric with a drop of glycerin, (**d**) para-aramid fabric with a drop of glycerin.

Contact angle measurements were performed to assess the hydrophilicity of the textile sample surface before and after modification, to check if the modification has an influence on this characteristic. The measurements were performed using a static method, which consisted of depositing a drop of test liquid on the sample surface and analyzing the drop shape using an optical system. Distilled water and glycerin with a volume of 5 μL were used as the test liquids. The contact angle was measured using an optical microscope at a room temperature of approximately 22 °C and a relative humidity of approximately 50%. Examples of the obtained images are shown in Figure 10.

In most cases, the water drop was immediately absorbed by the surface of the material (both modified and unmodified), which prevented reliable measurement of the contact angle—this indicates high wettability and sorption capacity of the fabrics. Only in the case of the para-aramid fabric was this process slightly slower. The contact angle was in the range of 100° to 110°. In the case of glycerin, the same situation occurred. Only in the case of para-aramid fabric was it possible to perform a reliable measurement, which are present in Table 4.

The measurement of the contact angle for the para-aramid knit fabric showed significant changes in the hydrophilicity of the surface depending on the modification stage. The clean sample was characterized by a relatively low contact angle of 35.95°, which confirms the moderate hydrophilicity of the material. Application of the paste containing expanded graphite before the ozonation process caused a further decrease in the angle to 23.52°, which may indicate the presence of surface groups or microstructure facilitating the spreading of the liquid. After application of the paste with EG after ozonation for 15 min, a slight increase in the angle to 27.42° was noted, but it remained at a level indicating the hydrophilic nature of the surface. However, a significant change occurred for the sample with the paste with EG after 30 min of ozonation—the contact angle increased to 52.41°, which suggests a deterioration in wettability. The obtained results indicate that the ozonation time of expanded graphite significantly affects the character of the surface of the composite material and should be carefully selected in the context of the planned functional applications.

### 3.4. Sensorics—Testing Sensitivity to Gases

**Figure 11:**
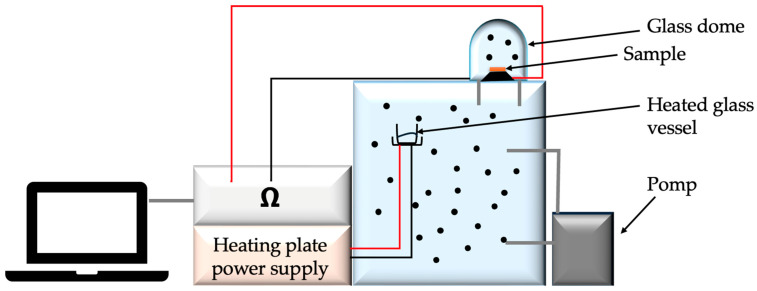
Measuring station for testing the sensory properties of manufactured textile gas sensors.

**Figure 12:**
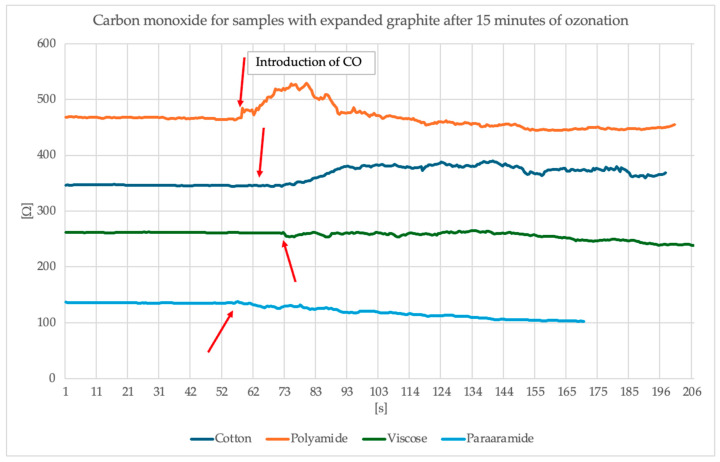
Graphs of surface resistance values during the carbon monoxide sensory testing for samples containing expanded graphite after 15 min of ozonation.

**Figure 13:**
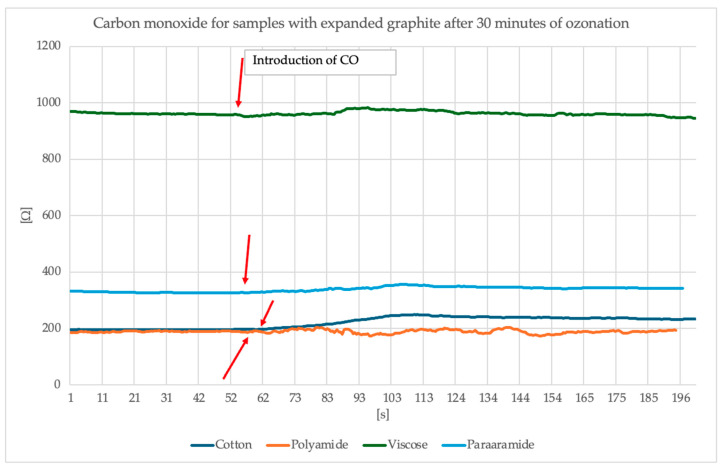
Graphs of surface resistance values during the carbon monoxide sensory testing for samples containing expanded graphite after 30 min of ozonation.

The measuring station for measuring changes in surface resistance under the influence of the presence of gases and chemical substances consisted of a glass chamber, a thermocouple and a thermohydrometer to maintain the temperature and air humidity inside the chamber at 23 °C and 25% relative air humidity, respectively, glass dome with electrodes connected to a Keithley multimeter and a pump injecting gas from the chamber into the dome. The study began with the introduction of a liquid into the glass vessel in the chamber, which, under the influence of the supplied heat, evaporated. After obtaining the appropriate gas concentration in the chamber, which was 100 ppm, the tested object was placed on the electrodes—in our case, a textile base with modification. Then it was covered with a dome. The multimeter was turned on. Readings were observed until the sample stabilized, and then the pump was turned on to supply gas to the dome while observing the changes in surface resistance. The sample was tested for approximately 60 s without any gas and 140 s with the gas inside the glass dome. After the test was completed, the next test object had to be inserted, while ensuring that the gas concentration in the chamber was at the appropriate level [31]. The measuring station is presented in Figure 11. Figure 12 and Figure 13 present the results of the study of the sensor response to carbon monoxide exposure.

Analysis of the surface resistance results presented above following exposure to carbon monoxide indicates that for samples in which graphite was exposed to 15 min of ozonation, the surface resistance exhibits a visible spike around the 60th second of exposure (the moment CO is switched on), particularly noticeable for the polyamide sample, where the resistance increased to over 500 Ω. Other materials (cotton, viscose, para-aramid) also responded, although the changes were less intense. Samples containing graphite ozonated for 30 min, on the other hand, exhibit a more stable and damp resistance profile. An overall improvement in signal stability is evident, and resistance values remain more uniform. In this case, the strongest response was observed for cotton, whose surface resistance values increased by approximately 50 Ω and stabilized at this level. However, for the other materials, the changes are smaller, more subtle or invisible. It is worth noting that despite the modification of the materials, different surface resistance values were obtained with the same paste.

To express the response of the sensors to gas, the sensory coefficient was numerically calculated according to the following formula:

(3) $$\frac{R_{0}-R_{k}}{R_{0}}\times 100\%$$

where $R_{0}$—initial resistance before gas introduction, $R_{k}$—final resistance after gas injection and shown in Table 5.

Analysis of the effect of ozonation time on the sensory properties of the tested materials revealed a clear variation in response depending on the fiber type. After 15 min of ozonation of the printing paste samples, the highest sensory index was recorded for para-aramid (31.07%), while cotton, polyamide and viscose reached values ranging from 9.66% to 12.07%. Extending the process to 30 min resulted in significant changes—cotton was the only one to significantly improve, increasing its parameter to 27.70%. A slight decrease was observed for polyamide (to 10.40%), while a significant reduction in sensory quality was observed for viscose (1.98%) and, especially, para-aramid (8.90%).

## 4. Discussion

The results obtained confirm the thesis that the use of the ozonation process in mild and controlled conditions can have a beneficial effect on the conductive properties of expanded graphite (EG). As shown in the work of Krawczyk and Skowroński, this process leads to the introduction of oxygen groups (such as hydroxyl, epoxy or carboxyl) to the graphite surface, which increases its electrochemical activity and improves its wettability and adhesion to substrates—features important in the context of sensor applications [32].

Based on the mechanisms described in the paper (particularly Section 1, Section 3.2 and Section 4), the impairment of conductive pathways due to excessive ozonation (30 min) involves three interconnected mechanisms:

1. Structural Fragmentation and Loss of Continuity
  - Prolonged ozone exposure causes over-oxidation of expanded graphite (EG), leading to severe fragmentation of graphene layers (Figure 2, Section 1).
  - SEM images (Figure 6d, Figure 7d, Figure 8d and Figure 9d) reveal the following:
  - Cracking, delamination and porous aggregates in the EG layer.
  - Discontinuous coverage of textile fibers (e.g., exposed fibers in viscose/para-aramid).
  - This disrupts the percolation network, forcing electrons to “hop” across gaps, increasing resistance.
2. Disruption of the π-Conjugated System
  - Ozonation introduces oxygen functional groups (e.g., epoxy, carbonyl, carboxyl) via oxidation (Equation (2), Section 1):
  - C_n_+O_3_ → C_n_O_x_
  - Short-term (15 min): Mild functionalization enhances hydrophilicity/adhesion without severely altering EG’s conductive sp^2^-hybridized carbon lattice.
  - Long-term (30 min): Excessive groups.
  - Convert conductive sp^2^ carbons to insulating sp^3^ hybrids.
  - Break the π-conjugated electron system (Section 4), reducing carrier mobility.

Too intense oxidation disrupts the π-conjugated electron system, increasing electrical resistance.

3. Degraded Adhesion and Layer Integrity
  - Over-oxidation makes EG hydrophilic and mechanically unstable.
  - Wettability tests (Table 2) show increased contact angles for para-aramid after 30-min ozonation (52.41° vs. 23.52° pre-ozonation), indicating reduced adhesion.
  - SEM confirms weakened EG-textile bonding (e.g., flaking in Figure 9d).
  - Fragile layers detach during handling/sensing, further breaking conductive paths.

Table 6 presents considerations related to the differences occurring at different ozonation times.

Key evidence from the paper

- Electrical Data: EG after 30-min ozonation exhibits higher surface resistance than 15-min samples (Table 3), e.g.,:
- Cotton: 0.568 kΩ (30 min) vs. 0.351 kΩ (15 min).
- Viscose: 1.249 kΩ (30 min) vs. 0.444 kΩ (15 min).
- Gas Sensing: Sensors with 30-min EG show a damped response to CO (Figure 12) due to reduced reactivity and conductivity.

Excessive ozonation fragments EG’s structure, converts conductive sp^2^ carbons to insulating sp^3^ domains, and degrades layer adhesion. This combination disrupts electron percolation, increasing resistance and reducing sensor performance. The 15-min “sweet spot” balances functionalization for adhesion while preserving conductive pathways. Importantly, with appropriately selected parameters, ozonation does not yet cause significant degradation of the crystalline structure of the material, thus avoiding excessive deterioration of electrical conductivity. In the presented studies, it was observed that the use of EG subjected to 15-min ozonation results in improved application properties of the paste and increased homogeneity of the deposit on the surface of the textile material. At the same time, surface resistance measurements showed favorable values of electrical conductivity compared to the paste containing non-ozonated graphite. It can therefore be concluded that the initial, moderate functionalization of the EG surface promotes improved current conductivity, probably due to better adhesion to the substrate and improved cohesion and continuity of the conductive layer. On the other hand, the results for the sample with the paste containing EG ozonated for 30 min indicate the opposite effect—conductivity deteriorated, and the surface structure became more porous and discontinuous, which can be associated with excessive functionalization leading to disruption of the continuity of conductive paths. This phenomenon is consistent with the mechanism described in the literature, where too intense oxidation of EG causes the disruption of the π-conjugated electron system and an increase in electrical resistance.

The results indicate that changes in the surface resistance of the samples in the presence of carbon monoxide result from interactions between CO and oxygen groups introduced to the surface of expanded graphite during the ozonation process. Ozonation leads to the formation of hydroxyl, carbonyl and carboxyl groups, which act as adsorption sites for CO molecules. This adsorption is associated with partial redox charge exchange and the introduction of additional electrons into the conductive network, which typically results in a decrease in surface resistance. In the case of samples prepared with graphite ozonated for 15 min, this effect is particularly pronounced, as the graphite structure maintains the continuity of the conductive pathways while simultaneously increasing the number of functional groups promoting CO adsorption. However, with longer ozonation (30 min), excessive oxidation leads to the formation of defects and disruption of the conductive network, which reduces the efficiency of electron transport and weakens the reaction in the presence of gas. Additionally, the observed differences between the textile substrates indicate that the morphology and wettability of the fibers influence the quality of the conductive coating and thus the intensity of the resistive signal in the presence of carbon monoxide. A similar mechanism of CO detection on the surface of carbon oxygen groups has also been described in the literature [33,34,35,36]

To sum up, the obtained results indicate that the EG ozonation process can be an effective tool for modifying its properties, if control over the reaction parameters is maintained. Short-term ozonation leads to improved conductivity and quality of coatings on textile substrates, while too long exposure times can have the opposite effect. In the context of designing active layers for sensor applications or functional conductive textiles, optimization of the ozonation process is a key step to obtaining the desired functional properties.

## 5. Conclusions

In the literature, one can read that the ozonation process does not have a positive effect on graphite. Ozone introduces functional groups (-OH, -COOH, -C=O), which change the hybridization of carbon, preventing electrons from moving freely, thereby reducing conductivity [16]. However, as demonstrated by the tests presented in this article, maintaining controlled and mild process conditions allows maintenance of or even improvement in electrical conductivity during ozonation of expanded graphite. The ozone concentration and time must be kept low. This results in the addition of a small number of functional groups while maintaining the crystalline structure of graphite. In our case, the ozonation process significantly improves the surface conductivity of the expanded graphite layer on all types of fabrics—probably due to the increase in the number of functional groups (e.g., carbonyl, hydroxyl), which improve the adhesion and distribution of the conductive material. The best results expressed in the lowest surface resistance were achieved after 15 min of ozonation. After 30 min, the parameters deteriorated. This indicates that the exposure time was too long. Para-aramid fabric and cotton knitwear show stable and beneficial properties both before and after ozonation, so they can be preferred substrates for textronic applications (e.g., resistive sensors).

After analyzing the images generated by the scanning electron microscope, it can be stated that the ozonation process significantly improves the adhesion of expanded graphite to the fibers, and the appropriately selected ozonation time—in our case, 15 min—allows for obtaining a continuous, uniform and well-adhering conductive layer. Too long ozonation—in our case, 30 min—may result in excessive degradation and loss of cohesion of the EG layer, which may negatively affect the durability of conductivity and mechanical properties of the material. The presence of pastes containing expanded graphite has a significant impact on the morphology of the surface of the materials. In each case, the use of paste with non-ozonated graphite led to clear coverage of the fiber surface with an even and compact layer of filler, which resulted in blurring the contours of the fiber and increasing the surface roughness. The use of expanded graphite previously subjected to 15-min ozonation resulted in a visible reduction in the coat density and partial exposure of the fiber structure—this phenomenon was particularly pronounced in the case of viscose and para-aramid fabrics. The most advanced morphological changes were observed in samples with graphite ozonated for 30 min, where the paste appeared in the form of dispersed aggregates with a porous structure, and the fiber surface was largely exposed. For para-aramid fabric, mechanical degradation and delamination were also noticeable. The obtained results clearly indicate that the ozonation process of expanded graphite affects its application and adhesion properties, reducing the ability to create uniform coatings on the surface of textile fibres. In addition, the effectiveness of the paste bonding with the substrate also depends on the type of fiber; natural and semi-synthetic materials show a different surface reaction than high-strength fibers. Finally, the graphite ozonation time should be carefully selected depending on the expected functional effect and the type of textile substrate used.

The contact angle measurement revealed very high liquid absorption by most of the tested materials, making it impossible to perform the measurement—the exception was the para-aramid knit, for which clear changes in the angle were noted depending on the modification stage. An important conclusion from this study is that materials modified with paste containing expanded graphite in various configurations do not change their hydrophilic and hydrophobic properties. These pastes have no effect on these characteristics. These results clearly indicate that the EG ozonation time significantly affects the surface and electrical properties of the composite, and the optimization of this process is a key element in the design of functional conductive textiles and active layers for sensing applications.

Based on the sensing measurements, a clear effect of expanded graphite ozonation time on the sensitivity of the textronic sensors to carbon monoxide can be observed. Different surface resistance values were also observed for the tested materials despite using the same modification and paste, suggesting that the properties of the textile substrate significantly affect gas detection. The curves for the samples modified with expanded graphite paste after 30 min of ozonation are stable throughout the experiment, despite the presence of gas after 60 s. This may indicate that longer ozonation reduces the graphite’s surface reactivity, resulting in lower sensor sensitivity but improved signal stability over time. The sensory index also indicated that the thirty-minute ozonation process did not have a beneficial effect on the expanded graphite in most cases, and the values obtained during this analysis were less satisfactory than those obtained for the fifteen-minute process. The only exception was cotton knitwear, for which an increase in the sensory index was noted. This may be due to the type of material and the binding of the printing paste to the knitwear. Now, the sensory coefficient is not fully satisfactory for the authors and work on its improvement will continue; however, the visible changes are very promising and motivate further use of this method.

In summary, the results suggest that expanded graphite ozonation time affects both the intensity of the response to CO and the nature of the resistance changes—a shorter time promotes higher reactivity, while a longer time improves stability but reduces the detection signal. Furthermore, the base material plays a significant role in modulating the electrical response of the system.

## Figures and Tables

**Table 1:** Comparison of the properties of graphite and expanded graphite [8,9,10].

Characteristic	Graphite	Expanded Graphite
Structure	Layered, flat sheets of carbon atoms	Extended, layers “stretched”
The process of receiving	Natural or synthetic (pyrolysis and graphitization)	Intercalation with acids and oxidants + rapid heating
Appearance	Heavy, gray or black, shiny	Light, voluminous, fluffy material
Density	~2.2 g/cm^3^	~0.01–0.05 g/cm^3^
Specific surface area	~10 m^2^/g	up to several hundred m^2^/g
Electrical conductivity	High	Good
Chemical resistance	Very good	Good

**Table 2:** Parameters of selected textile materials.

Textile Material	Surface Mass [g/m^2^]	Standard Deviation [g/m^2^]	Thickness [mm]	Standard Deviation [mm]	Air Permeability [mm/s]	Standard Deviation [mm/s]
Cotton knit	25.5	0.30	0.85	0.02	83.65	3.00
Polyamide knit	22.4	0.54	0.57	0.01	91.27	3.57
Viscose fabric	11.8	0.17	0.28	0.02	328.20	35.90
Para-aramid fabric	18.4	0.23	0.40	0.02	194.90	16.36

**Table 3:** Surface resistance results for screen-printed sensors with expanded graphite before and after ozonation process.

Textile Material	Surface Resistance [kΩ]					
	Expanded Graphite Before Ozonation	Standard Deviation	Expanded Graphite After 15 min Ozonation Process	Standard Deviation	Expanded Graphite After 30 min Ozonation Process	Standard Deviation
Cotton knit	120	128	0.351	0.130	0.568	0.473
Polyamide knit	1288	1379	0.950	0.461	0.248	0.247
Viscose fabric	1320	1595	0.444	0.162	1.249	0.558
Para-aramid fabric	174	113	0.157	0.662	0.223	0.130

**Table 4:** Contact angle values for para-aramid fabric before modification, with EG paste before ozonation, with EG paste after 15 min of ozonation, and with EG paste after 30 min of ozonation.

Paraaramid Knit	Wet Angle [°]	Standard Deviation [°]
Pure sample	35.95	3.60
With EG paste before ozonation	23.52	2.53
With EG paste after 15 min of ozonation	27.42	2.80
With EG paste after 30 min of ozonation	52.41	4.92

**Table 5:** Sensory coefficient results for the manufactured gas sensors.

Samples After 15 min of Ozonation	Sensory Coefficient [%]	Samples After 30 min of Ozonation	Sensory Coefficient [%]
Cotton	11.60	Cotton	27.70
Polyamide	12.07	Polyamide	10.40
Viscose	9.66	Viscose	1.98
Paraaramide	31.07	Paraaramide	8.90

**Table 6:** Why 15 min works vs. 30 min fails.

Parameter	15-min Ozonation	30-min Ozonation
Functionalization	Optimal O-groups improve adhesion	Excessive O-groups fragment EG
Conductive Paths	Intact sp^2^ network	Disrupted π-conjugation
Layer Morphology	Uniform, adherent coating (SEM)	Porous, cracked aggregates
Surface Resistance	Lowest values (Table 3)	Higher than 15-min samples

## Data Availability

To share research data, please contact the authors of the article by email.
